# Dynamic conversion of cell sorting patterns in aggregates of embryonic stem cells with differential adhesive affinity

**DOI:** 10.1186/s12861-020-00234-0

**Published:** 2021-01-06

**Authors:** Jeffrey D. Tse, Robert Moore, Yue Meng, Wensi Tao, Elizabeth R. Smith, Xiang-Xi Xu

**Affiliations:** Sylvester Comprehensive Cancer Center, Department of Cell Biology, Cell and Developmental Biology Graduate Program, University of Miami School of Medicine, Miami, FL 33136 USA

**Keywords:** Embryoid body, Embryonic stem (ES) cells, Cell sorting, Morphogenesis, Cell spontaneous assembly, Embryonic development, Adhesive, E-cadherin, Differential adhesive hypothesis, Apical polarity, Self-assembly, Differential adhesion hypothesis

## Abstract

**Background:**

Mammalian early development comprises the proliferation, differentiation, and self-assembly of the embryonic cells. The classic experiment undertaken by Townes and Holtfreter demonstrated the ability of dissociated embryonic cells to sort and self-organize spontaneously into the original tissue patterns. Here, we further explored the principles and mechanisms underlying the phenomenon of spontaneous tissue organization by studying aggregation and sorting of mouse embryonic stem (ES) cells with differential adhesive affinity in culture.

**Results:**

As observed previously, in aggregates of wild-type and E-cadherin-deficient ES cells, the cell assemblies exhibited an initial sorting pattern showing wild-type cells engulfed by less adhesive E-cadherin-deficient ES cells, which fits the pattern predicted by the differential adhesive hypothesis proposed by Malcom Steinberg. However, in further study of more mature cell aggregates, the initial sorting pattern reversed, with the highly adhesive wild-type ES cells forming an outer shell enveloping the less adhesive E-cadherin-deficient cells, contradicting Steinberg’s sorting principle. The outer wild-type cells of the more mature aggregates did not differentiate into endoderm, which is known to be able to sort to the exterior from previous studies. In contrast to the naive aggregates, the mature aggregates presented polarized, highly adhesive cells at the outer layer. The surface polarity was observed as an actin cap contiguously spanning across the apical surface of multiple adjacent cells, though independent of the formation of tight junctions.

**Conclusions:**

Our experimental findings suggest that the force of differential adhesive affinity can be overcome by even subtle polarity generated from strong bilateral ligation of highly adhesive cells in determining cell sorting patterns.

**Supplementary Information:**

The online version contains supplementary material available at 10.1186/s12861-020-00234-0.

## Background

The understanding of the basic principles in embryonic structure formation holds profound implications and potential to provide insights into the mechanisms of organogenesis and for application in tissue engineering and regenerative medicine [1, 23]. As such, biological axioms driving cell sorting and patterning during early embryogenesis represent a recurring focus of research in developmental biology.

The growth and development of murine blastocysts provides a relatively simple model for understanding embryonic cell proliferation, differentiation, and morphogenesis [3, 9, 14–16]. Blastocysts are formed upon the divergence of the equipotent morula cells into the first two cell lineages, toward either the trophectoderm or inner cell mass [9, 24]. The ensuing cell fate commitment occurs within the inner cell mass and specifies the primitive endoderm that forms an epithelial layer covering the epiblast lineages [12, 14–16, 21, 28, 36].

Various aspects of the developmental processes in the early mouse embryos can be replicated in culture by the embryoid body model, in which the aggregation of embryonic stem cells leads to proliferation, differentiation, and spontaneous morphogenesis [7, 11, 28, 43]. The embryoid body models are able to accurately replicate the in vivo biology of gene functions involved in early embryonic differentiation and morphogenesis [7, 11, 43]. Dab2 deletion results in the mixing of endoderm cells with epiblast cells in both embryos and embryoid bodies [33, 56, 57]. The beta1 integrin-deficient primitive endoderm cells segregate from, rather than form, a layer covering the epiblast in both embryos and embryoid bodies [35]. Deficiency in endoderm differentiation was found in both embryos and embryoid bodies of GATA6 [4, 6, 30, 45] or Grb2 [8, 10, 55] null genotypes. Pten is required for cavitation in both embryos and embryoid bodies [29].

Apparently, the ability of the early embryonic cells to spontaneously associate, differentiate, and sort to assemble tissue structures is programmed in the genome without environmental instruction. Townes and Holtfreter first pioneered insights into the ability for spontaneous assembly of early embryonic cells by demonstrating that dissociated embryonic amphibian cells can re-aggregate, self-assemble, and self-organize into configurations that resemble the original, discrete tissue anatomy [49, 54]. The chemistry term *affinity* was applied to their observations in order to encompass the combined attractive and repulsive forces occurring between cells as well as the segregation and patterning of cell types during development [54]. Alternatively, Malcolm Steinberg postulated the differential adhesion affinity hypothesis, now a well-known paradigm that asserts cells assembly according to adhesive strength to achieve the lowest entropy [46–48]. In such a pattern, the less adhesive cells migrate to the periphery of a heterotypic cell aggregate to consequently surround cells of higher adhesive affinity, thereby achieving the most thermodynamically stable configuration of the theorized closed cell system. While Steinberg’s differential adhesion hypothesis does offer a well-accepted, physics-modeled principle influencing cell sorting, the concept does not entirely clarify the underlying biological mechanisms of morphogenesis and embryogenesis. In fact, compelling evidence indicates other cellular properties such as such as metabolic energy, ability to form polarity, etc., may supersede adhesive affinity in dictating a heterotypic aggregate pattern [1, 20, 32, 34]. Since then, considerable interest and effort have been devoted to study the simple sorting of two cell types, and substantial understanding has been achieved [1, 5, 17, 20], though the questions have not been conclusively settled.

The primary molecule mediating intercellular adhesion in early embryo morphogenesis is E-cadherin [24, 27, 42, 50]. N-cadherin (or, neuronal cadherin) only has a small contribution to cell-cell adhesive affinity in the early mouse embryos [34]. When E-cadherin null embryonic stem (ES) cells were mixed and allowed to sort with wild-type ES cells, the sorted pattern conformed to the differential adhesion hypothesis --- the less adhesive E-cadherin null cells initially sorted to envelop the more adhesive wild-type cells [32]. However, upon retinoic acid-induced differentiation of only the more adhesive wild-type half, the subsequent intermixing with undifferentiated E-cadherin null ES cells yielded the opposite sorting pattern, where the differentiated, E-cadherin-expressing wild-type cells established the outer layer over the less adhesive, undifferentiated inner component [32]. The study indicates that cell polarity overcomes differential adhesive affinity for surface positioning. Furthermore, when ES cells deficient in either E-cadherin or N-cadherin aggregate to form chimeric assemblies and then allowed to sort out spontaneously, the two weakly interacting cell types segregate but fail to envelop the other cell type concentrically [34]. Moreover, heterotypic cell aggregates comprising of wild-type and slightly less adhesive N-cadherin null ES cells did not demonstrate the segregated sorting pattern predicted by the differential adhesion hypothesis but rather resulted in a stochastic, intermixed cell distribution. The result indicates that there is a threshold in adhesive difference of two cell populations to trigger cell sorting [34].

Previously we have studied and reported the sorting patterns of undifferentiated and differentiated, high adhesive and low adhesive (E-cadherin deletion) ES cells [32, 34]. Since then, we observed experimental results that differed from our previously documented cell sorting patterns, and we found a cell sorting pattern contradictory to that predicted by the differential adhesion affinity hypothesis [46–48]. This prompted us to further analyze the mechanisms of cell sorting and spontaneous pattern formation to resolve the unexpected findings by more extensively analyzing cell sorting using time-lapse video microscopy.

## Results

### Differential adhesive affinity and aggregation of wild-type and E-cadherin null embryonic stem cells

**Fig. 1:**
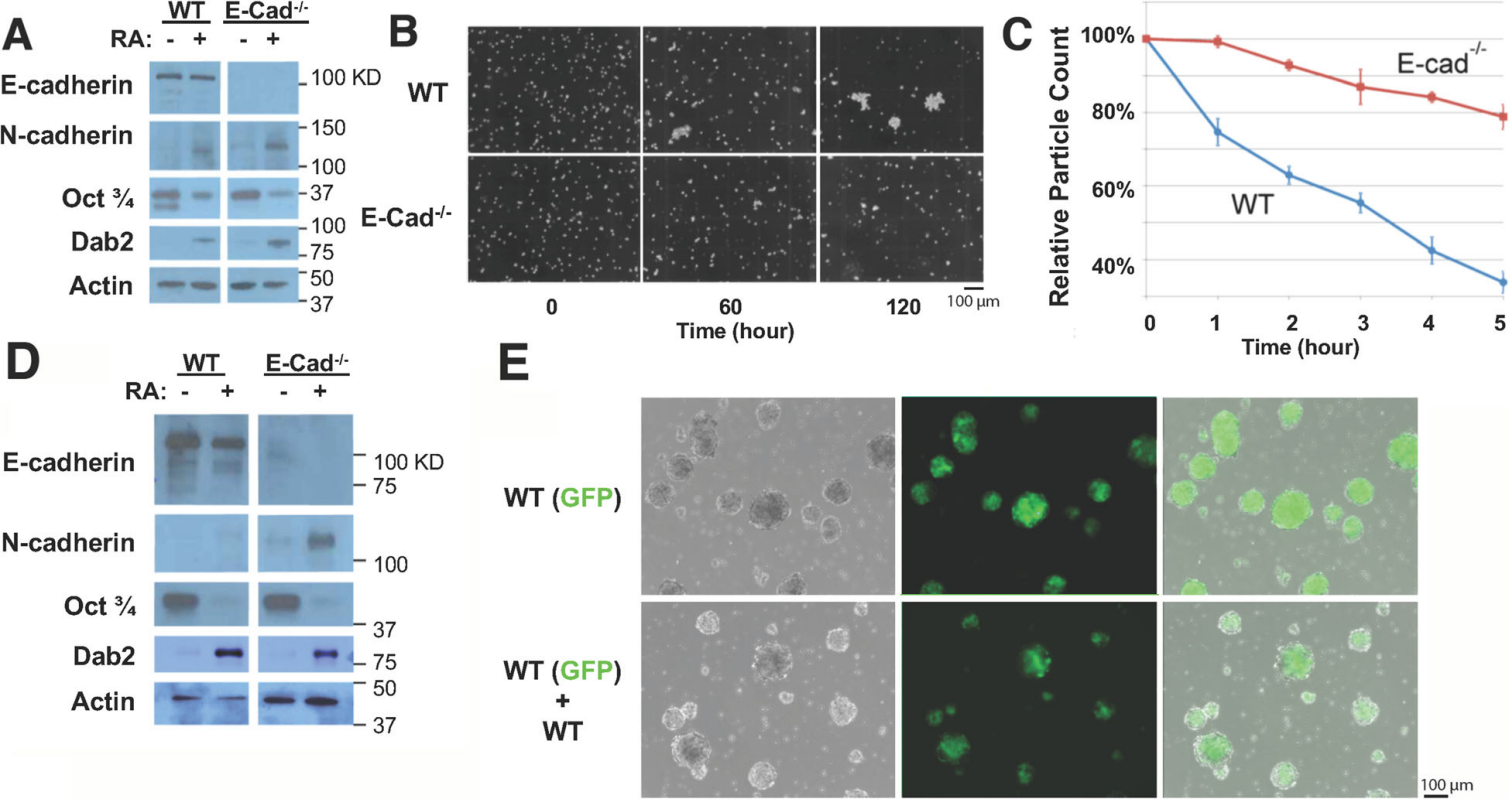
Characterization of wild-type and E-cadherin null ES cells. **a** Wild-type (WT) and E-cadherin null (E-cad (−/−)) ES cells in monolayer cultures with and without retinoic acid (RA) exposure were analyzed by Western blot for relative protein expression levels of the cell-cell adhesion molecules, E-cadherin and N-cadherin, as well as the pluripotency and endodermal differentiation markers, Oct3/4 and Dab2, respectively. Cell differentiation was achieved by treatment of the respective monolayer cultures with1 μM RA for 4 to 5 days. **b** Bright field images of homotypic aggregation of wild-type or E-cadherin (−/−) cells over a two-hour time course. **c** Cell adhesive affinity measured by aggregation rates of single cell suspensions of wild-type and E-cadherin (−/−) cells. The aggregation of the homotypic cell suspension yielded a serial reduction of particle count, measured using a Coulter Counter, over the time course. **d** Wild-type and E-cadherin null ES cell aggregates, cultured in suspension, without and with retinoic acid exposure were analyzed by Western blot for relative protein expression levels of E-cadherin, N-cadherin, Oct3/4, and Dab2, respectively. **e** Epifluorescence images depicting the control, 2-day cultured homotypic wild-type cell aggregates. The top row of images demonstrates the absence of non-GFP-labeled cells within the aggregates of WT-GFP cell culture. The bottom images establish intermixing of both WT-GFP and WT cells within the heterotypic cell aggregates

Following up our previous studies [32, 34], we used mouse ES to study cell sorting patterns in aggregates/embryoid bodies. Three ES cell lines, RW4 wild-type (WT), CFG37 GFP-labeled wild-type, and 9j E-cadherin null (E-cad (−/−)) cells were used in cell aggregation and sorting experiments. CFG37 ES cells were isolated from blastocysts from transgenic mice expressing GFP-histone H2B driven by the beta-actin promoter [32, 34, 40], and 99% of the cell population was GFP-positive with largely uniform signals in individual cells. Western blot analyses of the cells in standard adherent culture indicated that the 9j ES cells lacked E-cadherin protein, and had slightly elevated N-cadherin, possibly as a result of compensatory expression for the loss of E-cadherin, though N-Cadherin levels were increased in both WT and E-cadherin null cells following differentiation induced by retinoic acid (Fig. 1a). The cultured ES cells remained undifferentiated as indicated by the expression of Oct3/4 and were differentiated following treatment with retinoic acid as indicated by Dab2 induction and Oct3/4 reduction. In comparison, the E-cad (−/−) cells aggregated at a lower rate than those of wild-type, as observed under a microscope to observe the clustering of the cells (Fig. 1b). The lower adhesive affinity of the E-cadherin-deficient cells was also demonstrated by using a Coulter counter to measure the progressively declining numbers of particles as the cells clustered (Fig. 1c).

In suspension cultures, the cells formed aggregates but they still showed similar characteristics of cadherin and marker expression as determined by Western blot (Fig. 1d). The aggregates consisting entirely of CFG37 cells showed a uniform GFP signal throughout the whole spheres, while we were able to distinguish GFP-positive and negative cells in spheroids mixed with GFP-labeled or unlabeled cells (Fig. 1e).

### Interaction and assembling of embryonic stem cells to form aggregates in suspension culture

In cell sorting experiments, two or more different cell types were first dispersed into single cells, intermixed at a 1:1 ratio, and then placed on non-adhesive plastic dishes to allow cell aggregation. We mixed the WT-GFP with unlabeled ES cells, either RW4 WT or the E-cadherin deficient 9j (E-cad (−/−)) lines, and observed their association and cell sorting within the spheroids formed.

By time-lapse video microscopy, WT-GFP + WT and WT-GFP + E-cad (−/−) cells exhibited rather different characteristics in clustering, sorting, and assembling into spheroids. For the WT-GFP + WT unlabeled cell intermix, suspended individual cells rapidly clustered and associated into spheres by 8 to 12 h, with GFP-positive cells intermixed. The majority (> 95%) of aggregates contained both GFP-positive and negative cells, though the ratio appeared somewhat variable. Initially, the spheroids formed by collecting single cells around, and subsequently enlarged presumably by cell doubling. Collisions and fusion of neighboring spheroids to form larger aggregates were also frequently observed (Supplemental movie 1).

The heterotypic, WT-GFP + E-cad (−/−) aggregates developed in a distinctive manner as observed in all cases. Typically, following initial cell congregation, a small cluster of GFP-positive cells formed, surrounded by a cloud of unlabeled, E-cad (−/−) cells. The loosely gathered E-cad (−/−) cells appeared to follow the movement of the GFP-positive core. Cohesive aggregates of GFP-positive and -negative spheroids developed slightly slower than the mixture of all wild-type cells, by an approximate 12-h lag time. In the heterotypic (E-cadherin positive and negative) aggregates that formed, the cells moved dynamically against each other in the spheroids, though GFP-positive cells appeared segregated from the start (Supplemental movie 2). When two spheroids collided and combined, the GFP-positive central cores appeared to fuse, segregated from the GFP-negative, presumably E-cadherin-deficient cells in the periphery (Supplemental movie 3).

### Two main diverse sorting patterns in aggregates of undifferentiated embryonic stem cells with high and low adhesive affinity

As we have previously reported [32, 34], when pluripotent ES cells of wild-type and less adhesive E-cadherin knockout were mixed to form aggregates, the less adhesive E-cad (−/−) cells sorted to the outer layer, enveloping the highly adhesive wild-type ES cells. We attributed the cell sorting pattern to the differential adhesive affinity hypothesis proposed by Steinberg [46, 47, 49]. Nevertheless, in the previous studies, we also found that differentiated ES cells sorted to the outer layer to form a polarized endoderm epithelial layer, and concluded that the ability of the differentiated cells to establish apical polarity overcomes differential adhesive affinity to ultimately be positioned peripherally [32, 34].

**Fig. 2:**
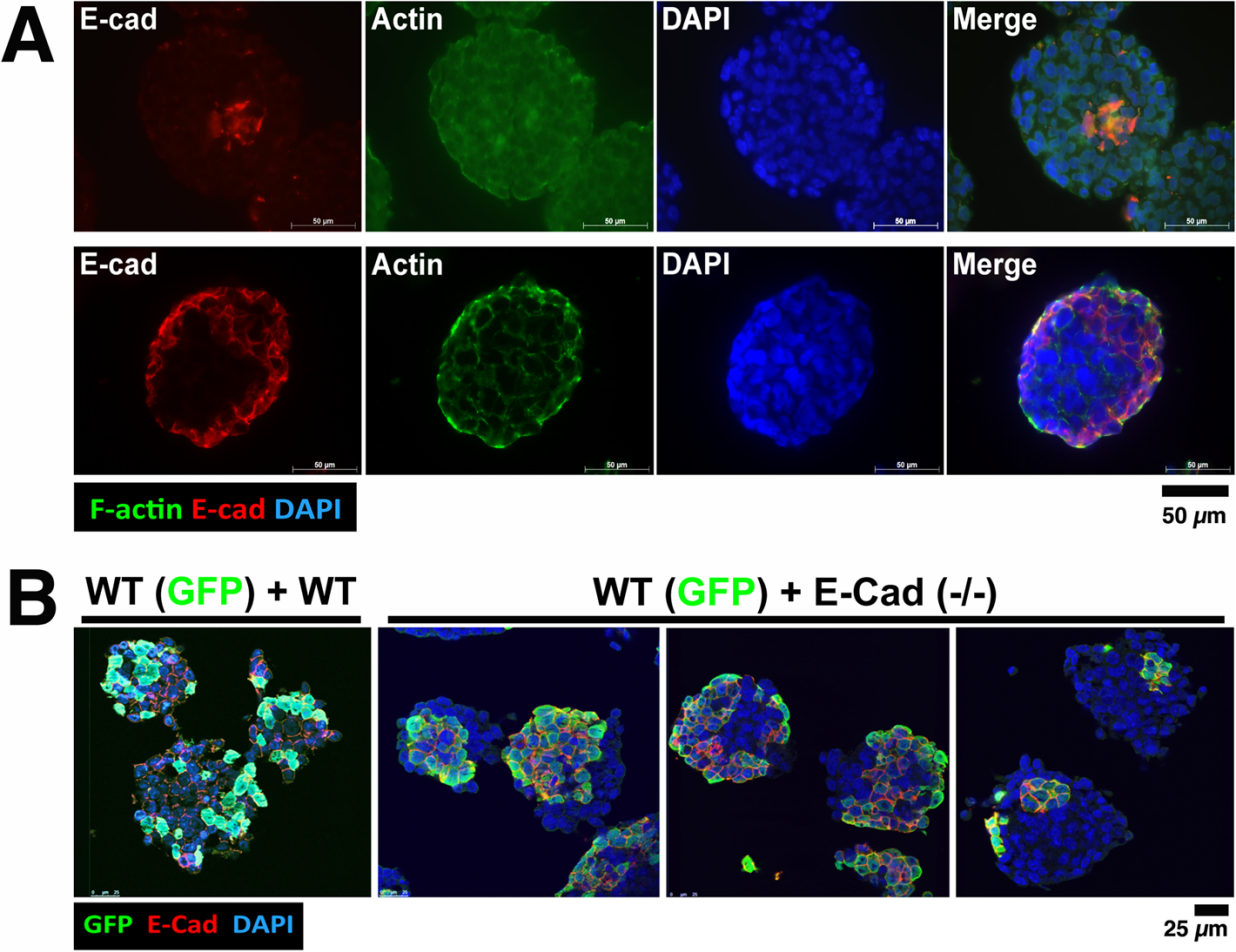
Sorting patterns in aggregates of wild-type and E-cadherin deficient ES cells. **a** Two representative cell sorting patterns: dispersed single cells from RW4 wild-type and E-cadherin-deficient 9j ES cells were mixed and allowed to aggregate for 2 days in suspension culture. The resulting cell aggregates were analyzed by histology, and 5 μm sections on glass slides were stained with E-cadherin and actin, countered stained with DAPI. Representative confocal images are shown for two main cell sorting patterns: either the wildtype (E-cadherin positive) cells were clustered in the center, surrounded by E-cadherin negative cells (upper panel); or the wildtype cells formed a shell on the surface, enveloping E-cadherin negative cells (lower panel). **b** Intermixed ES cells, either RW4 wildtype plus CFG37 (GFP-labeled WT), or the E-cadherin deficient 9j plus GFP-labeled WT, were allowed to aggregate in suspension culture for 2 days. Cryo-sections of the spheroids were analyzed by GFP epifluorescence, immunostaining of E-cadherin, and countered staining with DAPI. Representative examples are shown: one image from WT + WT-GFP, and three images from E-cadherin (−/−) + WT-GFP spheroids. Each sorting patterns ranked from 10 to 70% of the aggregates, variable in each independent experiment performed

In further reiterating the cell mixing and sorting experiments, however, we now found unexpected cell sorting patterns that diverged from the previously established conclusion [32, 34]. In some cases, the wild-type ES cells were found at the outer layer with the less adhesive E-cadherin null ES cells positioned in the interior (Fig. 2a**,** lower panel), in addition to the typical patterns (Fig. 2a**,** upper panel) reported previously. Here, immunostaining of E-cadherin was used to identify E-cadherin-positive and negative cells. For the mixture of wild-type and E-cadherin null cells (WT (GFP) + E-cad (−/−)), two representative contradictory examples are present: one showed that a shell of E-cadherin-positive cells enveloped E-cadherin negative cells (presumably E-cad (−/−)); the other showed a pattern in which E-cadherin-positive cells were centrally located surrounded by E-cadherin negative cells (Fig. 2a). The aggregates were produced by mixing undifferentiated wild-type and E-cadherin null ES cells and cultured for 2–4 days. In such a time frame, a negligible number of the ES cells underwent differentiation, which commonly initiates after day 4–5 of aggregation, as we have previously documented [7, 43].

To clarify the surprising observations, we further investigated the cell sorting patterns by mixing GFP-labeled cells with unlabeled cells to form aggregates and by performing live cell imaging and histology analyses. We compared immunostaining with the endogenous GFP signal of the labeled cells, and found that both E-cadherin immunostaining and GFP signal were equivalent and could distinguish E-cadherin wild-type and null cells (Fig. 2b). In these aggregates that were thought to be generated with a similar procedure, several sorting patterns were observed and documented (Fig. 2b). The mixing of WT and WT-GFP cells produced a largely random intercalated pattern, but mixing of WT-GFP and E-cadherin deficient ES cells generally resulted in a segregated configuration (Fig. 2b). As shown in 3 representative examples, the E-cadherin and GFP-positive cells sorted either to the center or periphery in individual aggregates. Moreover, in the third example, both surface and internally localized E-cadherin and GFP-positive cells were also present simultaneously in the same spheroids (Fig. 2b**,** right panel). From observations in 8 independent experiments, each of the three sorting patterns shown (Fig. 2b) could be found in the range from 10 to 70% among all the aggregates, indicating high inter-experiment variation in the resulted sorting patterns. We now realized that the variability of the sorting patterns was caused by the dynamic transition of the cell aggregates at the moment when the experiments were conducted and completed, and a slight difference in cell aggregation time can produce a large variation in cell sorting result.

### Initial sorting and subsequent maturation of aggregates of embryonic stem cells with differential adhesive affinity

**Fig. 3:**
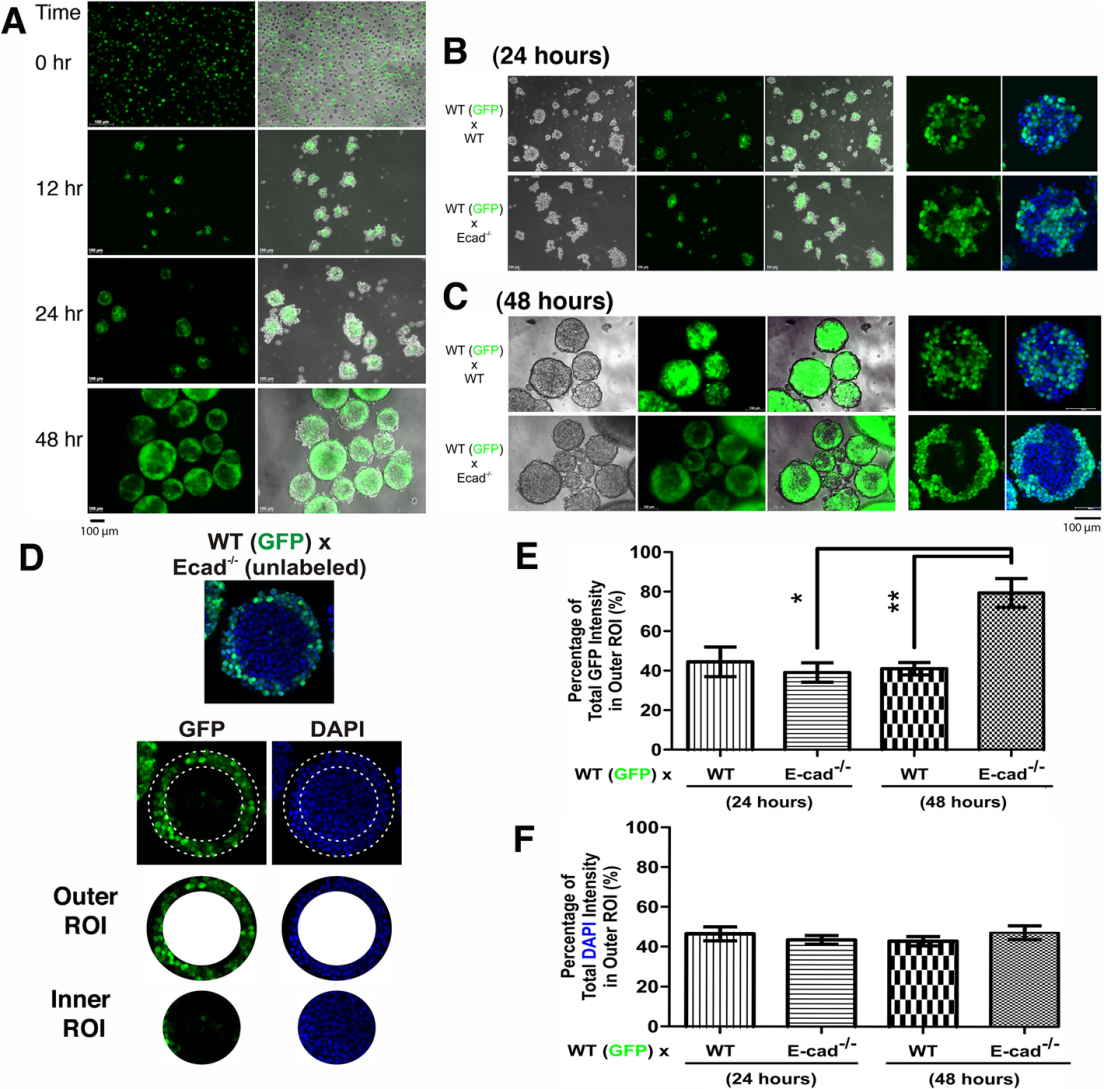
Time course for aggregation and maturation of spheroids. **a** Dispersed WT-GFP plus unlabeled E-cadherin-deficient ES cells were mixed and allowed to aggregate in suspension culture. Representative images of GFP signals and overlayed on bright field were shown for 0, 12, 24, and 48-h following mixing. **b** Images of 24-h spheroids from WT-GFP plus unlabeled wild-type or E-cadherin-deficient ES cells are shown. Optical sectioning by confocal microscopy of representative individual spheroid was also shown. **c** Images from 48-h spheroids are shown. Scale bar denotes 100 μm. The Z-stack confocal sectionings of the aggregates are included as supplementary results (Supplemental movie 3, 4, 5, 6). **d** An example shows the approach to quantitate the distribution of cell types in an aggregate from WT-GFP plus E-cadherin (−/−) ES cells. The image of the aggregate was divided into equal areas of outer ring or inner circle, defined as the region of interest (ROI). The intensities of GFP or DAPI in the ROI were quantitated by Image J program. **e** The distribution of GFP signal in the outer ROI (region of interest) was calculated and averaged from multiple aggregates, with standard errors indicated. The numbers of aggregates analyzed were 59 and 228 (wildtype), and 39 and 121 (wildtype plus E-cadherin null), for 24- and 48-h timepoint respectively. The differences are statistically significant by Student’s t-test, indicated by “*” and “**” for a *p* value < 0.005 in both cases. **f** The distribution of DAPI signal was shown as controls and for comparison

Following multiple repetitions of cell sorting experiments with intermixing of WT and E-cadherin null ES cells, we concluded that the highly adhesive WT ES cells unequivocally sorted initially to the interior of the cell aggregates as reported previously [32, 34]; however, upon subsequent maturation of the aggregates the E-cadherin-positive cells then localized to the surface. In a standardized protocol followed in the lab with precise cell density and mixing speed, we consistently observed that at an earlier time course (12 h) when cell aggregates were relatively small, the highly adhesive GFP-positive WT cells clustered in the center and were surrounded by unlabeled E-cadherin-deficient ES cells (Fig. 3a). By 24 h, both central and peripheral sorting patterns for GFP-positive cells were present (Fig. 3a). Finally, after 48 h in culture, the majority of GFP-positive cells localized as a shell enveloping the GFP-negative, presumably the E-cadherin-deficient, ES cells (Fig. 3a). Representative examples of optically sectioned 24-h (Fig. 3b) and 48-h (Fig. 3c) individual spheroids were analyzed, comparing heterotypic intermixing of WT-GFP plus WT controls with WT-GFP plus E-cad (−/−) ES cells (Supplemental movie 4, 5, 6, 7). The confocal sectioning of the aggregates provided visualization of the 3-dimensional distribution of GFP-positive cells within the spheroids (Supplemental movie 4, 5, 6, 7). The cell aggregates at 12 and 24-h time points appeared to harbor a rough surface, and the spheroid became progressively larger and rounder, with a smoother edge by 48 h (Fig. 3).

The relative location and distribution in the aggregates of GFP-labeled cells was quantitated by an image analytical approached we designed (Fig. 3d). GFP signals within individual aggregates were determined in equal areas of outer ring or inner circle, defined as the region of interest (ROI) (Fig. 3d). The results indicate that the GFP-positive cells relocated to the outer layer by 48 h in the aggregates composed of WT-GFP and E-cad (−/−) ES cells (Fig. 3e), though about equal DAPI signals, indication of cell number, were assessed (Fig. 3f). However, this analytical method did not show a distinct, central distribution of the WT-GFP in the 24-h aggregates mixing with the E-cad (−/−) ES cells, as the percentage of the GFP signals measured was not significantly lower in the outer ring (Fig. 3e). Although we did observe that the WT-GFP cells were more self-aggregated/associated in the mixtures with the E-cad (−/−) cells than with the unlabeled WT cells (Fig. 3b, c), indicating segregation of the two cell types with differential adhesive affinity. We reasoned that this was due to the fact that the E-cadherin and GFP-positive cells located both peripherally and interiorly, but superficially, however the cells were not necessarily at the central area of the spheres. Additionally, the internal to peripheral transition of the GFP-positive high adhesive wildtype ES cells likely initiated in some of the aggregates. We were unable to use this quantitative approach to satisfactorily analyze cell sorting pattern at 12-h time point because the aggregates were not spherical.

Nevertheless, these observations indicate that the maturation of cell aggregates correlated with the reversion of the initial cell sorting pattern of the mixtures of cells with differential adhesive affinity, the WT-GFP and E-cad (−/−) ES cells.

### Rapid transition of the sorting patterns

**Fig. 4:**
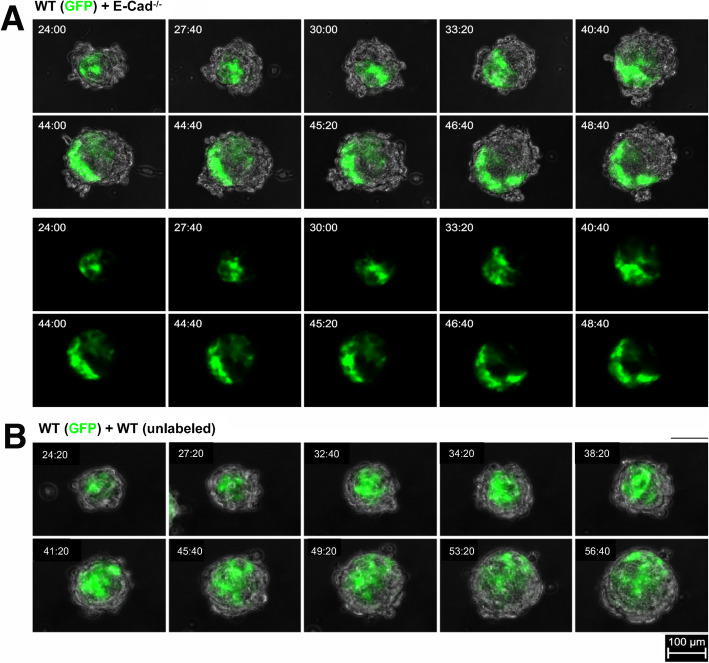
Transition of sorting patterns. Mixtures of GFP-labeled wild-type and unlabeled wild-type or E-cadherin deficient ES cells were cultured in suspension for 24-h to produce aggregates. Individual spheroids were then imaged by time-lapse video microscopy for another 24 to 48 h. **a** Serial images at about 3-h intervals were captured from a time-lapse movie of a representative heterotypic aggregate (starting at 24 h), composed of GFP-labeled wild-type and unlabeled E-cadherin (−/−) cells. Images of both GFP alone and overlaid on bright field are shown. **b** Serial images of an aggregate (starting at 24 h) composed of GFP-labeled and unlabeled wild-type ES cells are shown as a control. The time-lapse movies are included as supplementary data (Supplemental movie 7, 8)

To investigate the transition of cell positioning patterns, we used time-lapse imaging to visualize the reversion of the cell sorting (Fig. 4a). GFP-labeled WT and unlabeled E-cadherin knockout ES cells were intermixed and allowed to coalesce in suspension to form aggregates for 24 h. The aggregates were analyzed for progressive changes using an enclosed, temperature-regulated epifluorescence microscope system with imaging at 20-min intervals for additional 24 to 48 h.

During the early time course, the predicted differential adhesive affinity pattern of heterotypic aggregates was observed with the GFP-expressing, highly adhesive WT cells in the core of the aggregate surrounded by the unlabeled, less adhesive, and peripheral E-cadherin knockout cells (Fig. 4a). Initially, both E-cadherin-positive and -negative cells actively moved against each other, though the segregation of GFP-positive and -negative cells was preserved. At around the 40-h time point, the GFP-positive cell cluster extended to the outer layer. Subsequently, a layer of the GFP-positive cells formed a partial surface on the spheroid, and the superficially positioned GFP-positive cells appeared to contact and bring additional associated GFP-positive cells to extend the surface shell (Supplemental Movie 8). After reaching the surface, the GFP-positive cells appeared to slow their motion, and the layer of GFP-positive cells was maintained and persisted for at least 8 h in the recording (Fig. 4a).

As a control, no sorting patterns or bleaching of GFP signals were observed in cell aggregates from mixing of the WT-GFP with WT ES cells (Fig. 4b) (Supplemental Movie 9). Thus, the observations using time-lapse imaging indicate that the transition of sorting patterns occurs rapidly, and the surface positioning of the highly adhesive cells is stable.

### Formation of polarized apical actin caps on the surface of mature aggregates

**Fig. 5:**
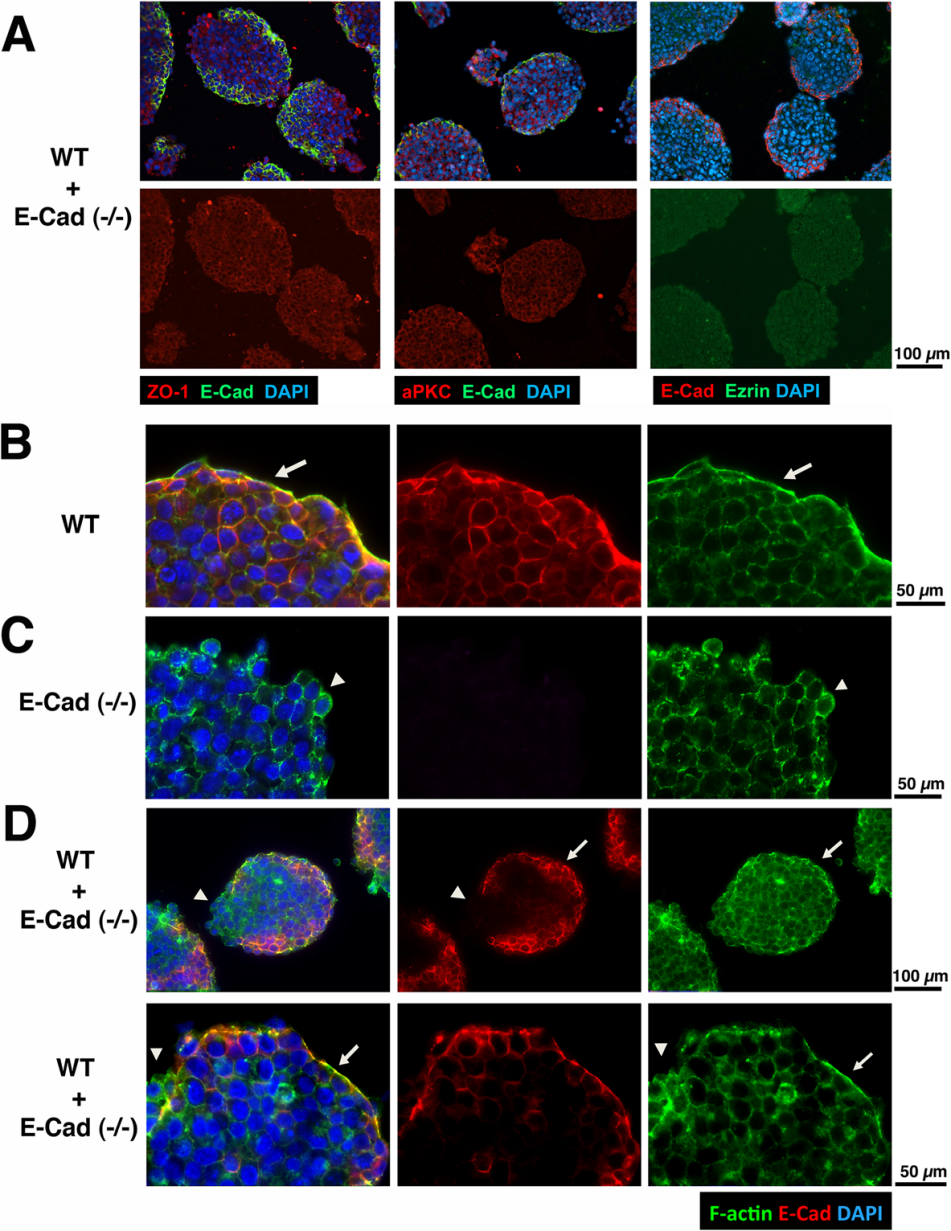
Polarity markers and differential distribution of cellular F-actin in ES cell aggregates. **a** Representative confocal immunofluorescence images of 2-day cultured aggregates from mixture of WT and E-cadherin null ES cells analyzed for E-cadherin and tight junction dependent classical apical polarity markers including ZO-1, aPKC, and Ezrin, and countered stained with DAPI. **b** Representative confocal immunofluorescence images of mature, 2-day cultured wild-type cell aggregates analyzed for E-cadherin, beta-Actin, and countered stained with DAPI. An arrow indicates the presence of apical actin staining on the surface of the spheroid. **c** Representative confocal immunofluorescence images of 2-day cultured E-cadherin null ES cell aggregates analyzed for E-cadherin, beta-Actin, and countered stained with DAPI. An arrowhead indicates that the actin staining of surface cells distributes rather uniformly around cell border, lacking polarity. **d** Two examples of spheroids derived from heterotypic mixing of wild-type and E-cadherin (−/−) ES cells were analyzed for confocal immunofluorescence staining of E-cadherin and actin, counter stained with DAPI. An arrow indicates the surface area that is E-cadherin-positive and shows a polarized actin cap. An arrowhead indicates surface region that is composed of E-cadherin null cells and contains uniformly distributed actin

The formation of polarity is a possible mechanism for the surface positioning of the cells, as in the case of primitive endoderm positioning on the surface. We first examined the mature (48 h) aggregates from the mixture of WT-GFP and E-cad (−/−) cells for the distribution of the classical polarity markers, ZO-1, aPKC, and Ezrin (Fig. 5a). In these aggregates, the wildtype cells located to the surface, as indicated by immunostaining of E-cadherin. However, no obvious diverged distribution of the classical tight junction associated polarity markers, ZO-1, aPKC, and Ezrin, was observed (Fig. 5a). As positive controls similar to that we reported previously, the polarized distribution of ZO-1 and aPKC was observed in the ES cell aggregates at a later time course, when surface extraembryonic endoderm developed or cavitation to form ectoderm initiated [29]. Thus, the re-distribution of E-cadherin expressing cells to the outer layer is not associated with formation of the classical tight junction dependent apical polarity of the surface cells.

Initially we observed that cell aggregates of the two contradictory cell sorting configurations exhibited very different F-actin staining patterns (Fig. 2a), and we suspected that the highly adhesive ES cells formed a polarized epithelium to be able to position on the surface. Thus, we further examined the distribution of F-actin in cell aggregates (Fig. 5b, c, d). In mature (48 h) spheroids derived from wildtype ES cells, the surface was covered with a layer of strong actin staining that consisted of multiple contiguous surface cells, suggesting the formation of a surface epithelium and consolidated apical actin organization (Fig. 5b, arrow). In contrast, cellular actin staining was uniformly distributed around surface cells of E-cad (−/−) ES cell aggregates that had not yet had sufficient time to fully develop and compact in the 48-h incubation time (Fig. 5c, arrowhead). In spheroids composed of mixed WT and E-cadherin deficient cells, an F-actin cap was observed on the surface that was composed of E-cadherin-positive cells (Fig. 5d, arrow), but not on the surface where E-cadherin-deficient cells localized (Fig. 5d, arrowhead), as shown in two examples. Thus, we conclude that the highly adhesive E-cadherin wildtype cells on the surface formed a polarized epithelium (as indicated by the distribution of beta-actin), which may account for the ability of the WT cells to sort to the surface and to envelop the less adhesive E-cadherin null cells.

### Polarization of a surface epithelium prior to differentiation in mature ES cell aggregates

Previously, we determined that differentiated ES cells in the aggregations containing undifferentiated cells were able to overcome the force of differential adhesive affinity to position on the surface [32]. The ability for the differentiated endoderm cells to position on surface was attributed to their propensity to establish an apical polarity facilitated by the Dab2-dependent endocytic trafficking [32, 33].

However, we reasoned that the presently observed cell sorting property of the highly adhesive ES cells to the surface was independent of endoderm differentiation, because extensive differentiation occurs only after 4 or more days of ES cell aggregation [7, 43]. To verify, we designed experiments to determine the relationship between the formation of a spheroid surface actin cap and endoderm differentiation.

**Fig. 6:**
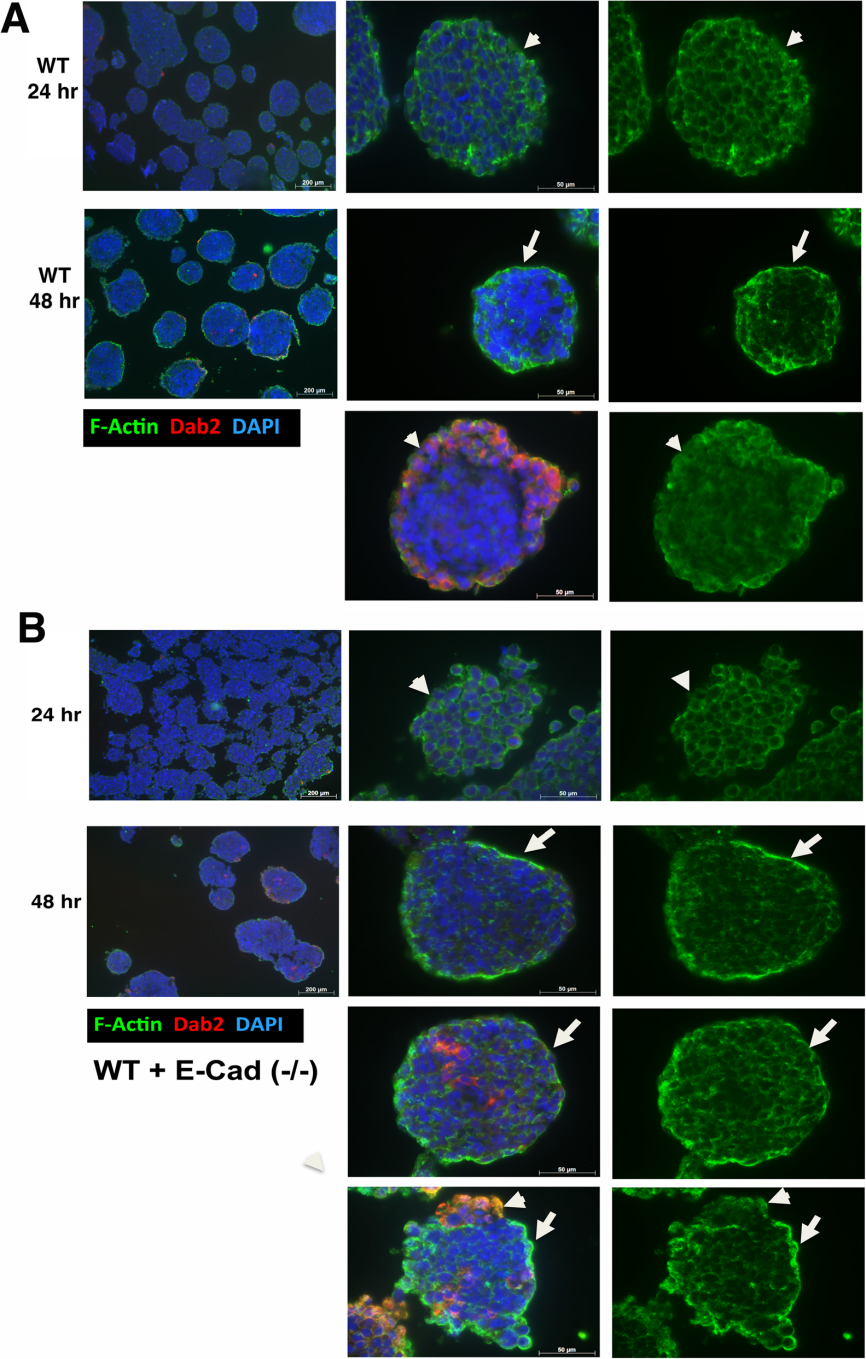
Formation of polarized surface is independent of endoderm differentiation in ES cell aggregation. **a** Spheroids from the 24- and 48-h aggregation of wild-type ES cells were analyzed for confocal immunostaining of Actin and Dab2 (a marker for endoderm differentiation). Individual spheroids are also shown at higher magnification. An arrowhead indicates surface cells with uniformly distributed actin at 24-h time course. An arrow indicates polarized actin cap on the apical domain of surface cells that are undifferentiated (Dab2-negative). In a rare spheroid containing differentiated cells, the differentiated cells positioned on the surface lack actin cap, as indicated by an arrowhead. **b** Confocal images of spheroids from aggregation of wild-type intermixed with E-cadherin deficient ES cells are shown for Actin and Dab2 immunostaining. Individual spheroids presented at a higher magnification show uniform Actin staining at 24 h (arrowhead), and polarized and undifferentiated superficial cells harboring actin cap at 48 h (arrow). Rare spheroids containing differentiated cells also show an actin cap on the apical surface of undifferentiated surface cells (arrow)

In the aggregation of wildtype ES cells, similar to that reported previously, no endoderm differentiation occurred within 24 h, as indicated by staining for the endoderm marker Dab2 [56, 57] (Fig. 6a). The cells located on the surface exhibited nearly uniform and diffused actin staining around the cell boundary (Fig. 6a, arrowhead). Few Dab2-positive cells were visible even within 48-h aggregates. In nearly all these 48-h, mature spheroids, an actin cap had formed on the surface (Fig. 6a, arrow). In rare spheroids in which a surface endoderm epithelium had formed, no actin cap was observed (Fig. 6a, arrowhead). The actin showed a dispersed staining pattern in the differentiated, Dab2-positive endoderm cells on the surface. Thus, the nature of apical polarity of the endoderm cells is distinctive from that of the undifferentiated surface cells signified with of an actin cap.

For aggregates composed of intermixing of wild-type and E-cadherin-deficient ES cells, the actin of the surface cells was also not polarized in 24 h (Fig. 6b, arrowhead). Most of the 48-h aggregates showed a partial actin cap though they contained no differentiated cells (Fig. 6b, arrowhead). Based on previous results (Fig. 5d), these cells possessing an actin cap are likely E-cadherin-positive rather than -deficient. In rare spheroids containing Dab2-positive cells either in the interior or on the surface, a partial actin cap and polarized cells were visible on the surface where Dab2 staining was absent (Fig. 6b, arrow). Consistently, the Dab2-positive endoderm epithelial cells positioned on the surface showed a diffuse beta-actin staining pattern (Fig. 6b, arrowhead).

Based on these observations, we conclude that a polarized epithelium forms on the surface of a mature spheroid without undergoing endoderm differentiation. We speculate that this polarity, signified by an actin cap on the apical surface of the adhesive E-cadherin-positive cells, accounts for the ability of the wild-type ES cells to sort to the surface, and to envelop the less adhesive E-cadherin-deficient cells.

## Discussion

In this study, our initial effort to clarify the observed diverse cell sorting patterns led to the discovery of a transitional state of cell polarization on the surface of aggregates of undifferentiated ES cells. The formation of such a subtle polarity is suggested to be the force responsible in reversing the cell sorting distribution predicted by Steinberg’s differential adhesion hypothesis [46, 47, 49]. Strategies will need to be developed to test the potential causative of the subtle apical actin cap polarity in positioning the highly adhesive cells on the surface. Remarkably, the current results indicate that a seemingly simple cell aggregation between two differentially adhesive cell types involves diverse and interesting cell sorting behaviors and complex underlying mechanisms.

**Fig. 7:**
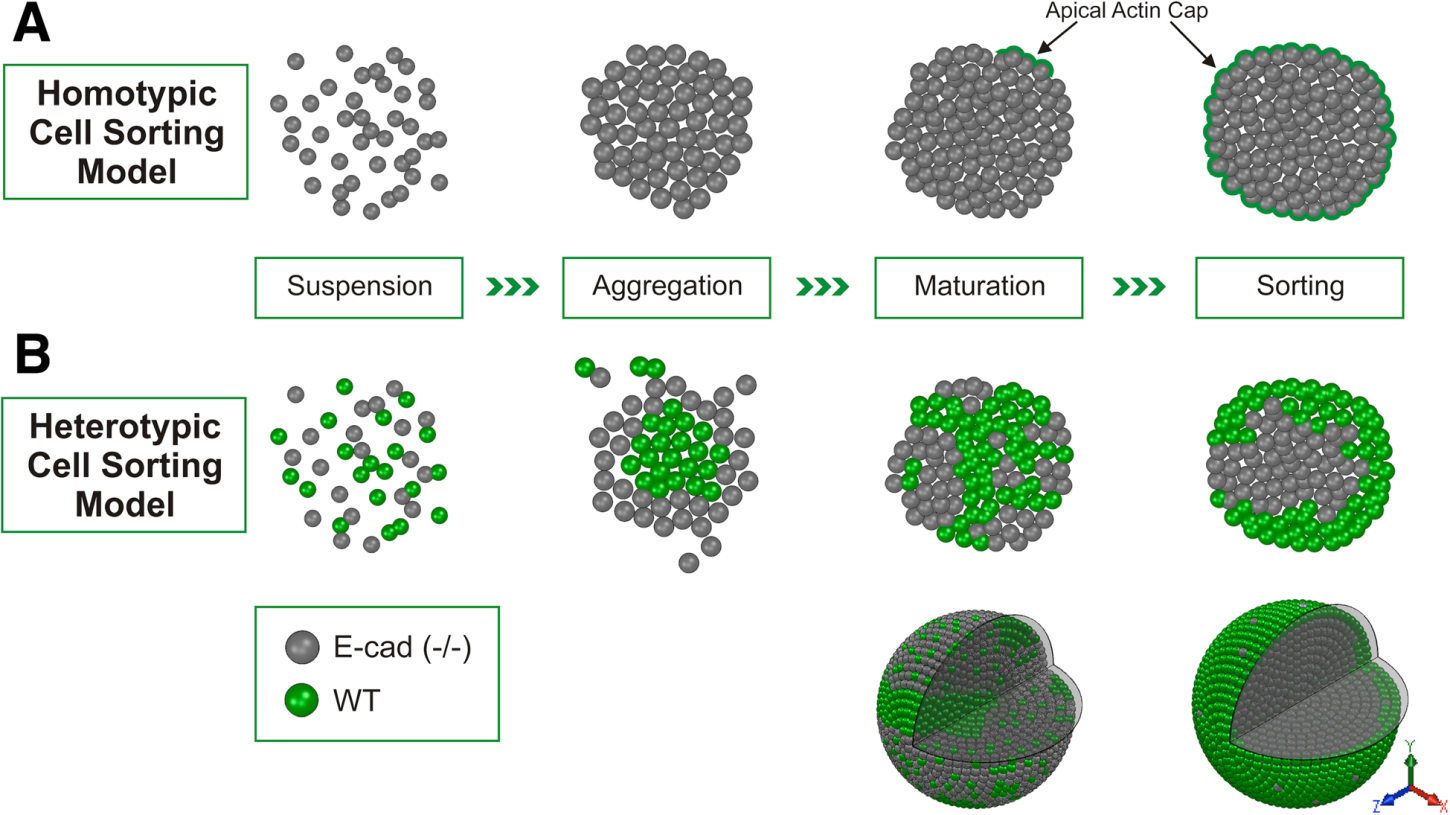
Proposed Models and Illustrations. **a** Maturation of ES cell aggregates coincides with the formation of a surface actin cap prior to differentiation. We discovered that ES cell aggregates mature with further culture and form a polarized surface that can be observed with a polarized actin cap, prior to the initiation of endoderm differentiation. **b** Mechanism for the reversion of cell positioning with adhesive affinity. When a highly adhesive cell type (such as wild-type ES cells) is intermixed with a less adhesive cell type (such as the E-cadherin deficient ES cells), the highly adhesive cells are sorted to the interior, enveloped by the less adhesive cells. The pattern is predicted by Steinberg’s differential adhesive affinity hypothesis. However, we observed that upon maturation, the highly adhesive cells subsequently form an outer shell, surrounding the less adhesive cells, a pattern contradictory to the differential adhesive affinity hypothesis. The ability for highly adhesive cells to form a polarized surface layer is a likely explanation for the observed cell sorting behavior that contradicts the differential adhesive affinity hypothesis. The process of cell sorting and positioning is illustrated, and 3D depictions of the different sorting patterns are generated based on optical sectioning of the representative spheroids

Our observations revealed that the aggregates of embryonic stem cells undergo maturation in culture prior to primitive endoderm differentiation, leading to the formation of a polarized surface composed of a contiguous layer of cells (Fig. 7a). This seems to be the result of consolidation and strengthening of E-cadherin-mediated adhesion of the surface cells. The E-cadherin-mediated strong adhesion then impacts cytoskeleton organization and leads to the formation of an observable actin cap on the apical surface. A role for cytoskeleton in breaking cellular symmetry and creating polarity is recognized [37]. For heterotypic aggregates with low and high adhesive cells, the initial configuration is that predicted by Steinberg’s differential adhesion hypothesis, according to which highly adhesive cells sort to the center and are surrounded by less adhesive cells (Fig. 7b). However, based on our current observation, we postulate that formation of an adhesive epithelium on the surface retains some of the highly adhesive cells to the outer layer. Eventually most of the highly adhesive cells migrate to the periphery by virtue of high cell-cell binding affinity, enveloping the less adhesive cells in the interior. This phenomenon represents another mechanism of cell sorting pattern that contradicts Steinberg’s differential adhesive hypothesis. The finding also emphasizes that subtle cell polarity may be able to overcome the configuration with the highest free energy (lowest entropy) provided by differential binding affinity [46, 47, 49], to dictate arrangement of cells with differential adhesive strength.

Previously, the dominance of cell polarity over differential adhesion in determining cell positioning was already noted in the organization of endoderm in murine embryoid bodies [32]. Upon lineage commitment of ES cells to primitive endoderm, the differentiated cells are able to establish epithelial polarity and position on the surface, through a Dab2-dependent process [33, 56, 57]. Dab2, an endocytic adaptor, is thought to generate apical polarity by enabling directional endocytosis and cargo transport [31, 52]. In either embryos or embryoid bodies derived from mice or ES cells of homozygous Dab2 gene deletion, the endoderm cells are not able to organize and position on the surface; rather, the cells intermix and distribute throughout without an organized pattern [33, 56, 57]. The formation of tight junctions and the polarized distribution of these markers were observed to closely associate with the sorting and development of primitive endoderm maturation cell surface [29, 44]. In embryoid bodies, distinctive distribution of aPKC and ZO-1 was observed at the apical surface when the polarized extraembryonic endoderm or the ectoderm layers were formed [29], indicating the formation of tight junctions is associated with the polarization of these epithelia.

In the current observation, the polarity and surface distribution of the highly adhesive cells appear to use a mechanism different from that employed by primitive endoderm, in which Dab2-mediated endocytic trafficking is required for apical cell polarity and surface positioning [33, 56, 57]. Also, the observed polarity of the surface cells here is independent of the formation of tight junction that can be observed by the polarized distribution of the classical markers such as ZO-1, aPKC, and Ezrin [29]. Particularly, the polarized actin cap forms prior to cell differentiation and Dab2 expression. The formation of an apical actin cap appears to require the adhesion of multiple neighboring cells to establish a contiguous epithelium, and the polarized actin cap spans multiple cells along their apical domains. In contrast, the Dab2-mediated endoderm polarization and surface positioning appear to be cell autonomous [32, 43]. This may indicate that modulating intercellular adhesiveness by way of E-cadherin expression facilitates both the polarization and movement of the cells being sorted to the surface. Not only do E-cadherins serve as cell adhesion molecules but they also possess the capacity to initiate local reorganization of the actomyosin and actin network, thereby entailing an induction of cell polarization [18, 19, 26, 27, 51].

Additionally, the cells harboring the apical actin cap are undifferentiated, and we did not observe expression and deposition of laminin or collagen IV to form a basement membrane. This polarized surface layer presumably can be punctured and displaced by primitive endoderm cells moving to the surface following their differentiation afterward. In contrast, endoderm cells express laminin and collagen IV, and assemble a basement membrane layer underneath as they position on the surface to form an epithelial layer [35].

## Conclusions

Since the experiments of Townes and Holtfreter that have now became content documented in textbooks [49, 54], the phenomenon and mechanisms of cell sorting and spontaneous assembly continue to gather interests. Numerous studies have addressed the topic by experimentation [22, 32–35, 38, 39, 43, 53] and conceptual consideration [1, 2, 5, 13, 17–20, 23, 27, 41, 53]. Here, we further examined ES cell organization by analyzing aggregation and subsequent configurations of mouse ES cells with differential adhesive affinities with respect to the intercellular adhesion molecule E-cadherin. We found that an initial segregated patterning with less adhesive E-cadherin-deficient ES cells enveloping wild-type cells transformed, upon further aggregate maturation, into a pattern with the adhesive wild-type element establishing an undifferentiated outer shell that enveloped a cluster of the less adhesive cells. These findings lead to the discovery of a transient state in which a polarized surface layer forms as ES cell aggregates mature but prior to endoderm differentiation (Fig. 7). The current finding of the ability of highly adhesive cells to form subtle actin cap and achieve surface positioning represents a new understanding into the basic principles of spontaneous cell sorting and self-assembling.

## Methods

### Embryonic stem cells: mutant and wildtype

RW4 (wild-type), CFG37 (wild-type cells that express the bACT-GFP transgene) [34, 40], and 9 J (E-cadherin homozygous null) [24, 25, 32] mouse embryonic stem (ES) cell lines were used in this study. All these cells were generated from blastocysts of mutant mice from our lab, and were reported previously [32, 34, 43]. The ES cells were maintained and expanded on feeder layers of irradiated mouse embryonic fibroblasts in ES cell culture medium (Dulbecco’s Modified Eagle Medium with 15% (v/v) fetal bovine serum, 2 mM L-glutamine, 1x mixture of nonessential amino acids, 50 mg/ml streptomycin, 50 IU/ml penicillin, and 0.1 mM beta-mercaptoethanol) supplemented with 1000 units/ml of recombinant LIF (ESGRO, Chemicon International) in a moisturized cell culture incubator at 37 °C and 5% CO_2_. Prior to experiments, the cells were harvested and re-plated on gelatin-coated tissue culture plastic plates without feeder cells. Inclusion of retinoic acid (1 μM) in culture medium for 4 days was applied to differentiate the ES cells into primitive endoderm like cells. Typically, more than 90% of the cells were differentiated as indicated by strong GATA4 and/or Dab2 expression, detected by immunofluorescence microscopy.

### Formation and culture of homotypic and heterotypic cell aggregates

The basic procedures for ES cell aggregation and embryoid bodies formation were similar to those described previously [32, 34, 43]. In the current study, a standardized procedure was used to minimize variations in timing, sphere size, and sorting patterns among individual researchers in the lab. The cell aggregate culture parameters had been determined to ensure that the resulting aggregates yielded relatively standardized sizes, similar in dimensions to an actual E5.5 embryo (approximately 100 to 200 μm in diameter). Briefly, cell aggregates were formed from 5 × 10^6^ dispersed and well mixed pluripotent ES cells in a 100 mm bacterial petri dish with 10 ml ES medium. Heterotypic aggregates were prepared by mixing equal numbers of two different ES cell types - one population fluorescently-labeled while the other was unlabeled. Cell numbers were determined by hemocytometer, Z2 Coulter Counter (Beckman Coulter) and Moxi Z Mini Automated Cell Counter (ORFLO). The cell mixture was allowed to coalesce in suspension. For the cell sorting experiments, cell aggregate culture medium contained LIF (1000 units/mL) to reduce undesired spontaneous differentiation.

### Antibodies, immunofluorescence microscopy, and Western blot

Primary antibodies used include: anti-E-cadherin (BD Biosciences, 610,181), anti-N-cadherin (BD Transduction Labs no. 610920), anti-Dab2 monoclonal (BD Biosciences, 610,465) and polyclonal developed [6], anti-beta-actin (BD Biosciences, 612,656), anti-Oct3/4 (Santa Cruz Biotechnology, sc-5279), anti-ZO1 (Invitrogen, Inc., #61–7300), anti-Ezrin (Abcam, [3C12] ab4069), anti-aPKC (Santa Cruz Biotechnology, In., sc-216), and anti-E-cadherin (BD Transduction Labs no. 610181).

For imaging and analyses with immunofluorescence microscopy, cell aggregates were fixed with buffered formalin, embedded in paraffin, and sectioned and placed on positively charged glass slides, as previously described [32, 34, 43]. Specimens on slides were deparaffinized in xylene, hydrated through a graded ethanol series, washed in water, and boiled in antigen retrieval solution (10 mM sodium citrate, pH 6.0). After blocking in 2.5% horse serum (Vector Laboratories), specimens were incubated in primary antibody solutions overnight at 4 °C, washed three times with PBS, then incubated with the corresponding specie-specific secondary antibodies. Multiple secondary antibodies conjugated with the respective Alexa fluorochrome were applied for simultaneous imaging of up to three antigens. DAPI (4′-6-diamidino-2-phenylindole) solution was applied as a nuclear counterstain prior to ProLong Gold Antifade reagent and coverslip mounting.

Images were captured with an inverted Zeiss AxioObserver Z1 operated by Axio Vision 4.8 software and a Plan-Apochromat 63X (oil immersion, N/A 1.4) or A-Plan 10X (N/A 0.25) objective mounted with a monochrome Zeiss AxioCam MRm CCD camera. Confocal imaging was performed with a Zeiss LSM510/uv Axiovert 200 M inverted, laser scanning confocal microscope operated by Zeiss LSM software. For live imaging, embryoid bodies were suspended in medium buffered with 10 mM HEPES, pH 7.4, and imaged in a glass bottom micro-well dish (MatTek Corporation, MA, USA) and the Plan-Neofluar 25X lens (water immersion, N/A 0.8).

For Western blot, following primary antibodies inculation, horseradish peroxidase conjugated secondary antibodies of goat or mouse origins (BioRad; Jackson Immunolab; Zymed) against corresponding primary antibodies were used. Chemoluminescence detection was achieved using Amersham ECL Western Blotting Detection Reagent (GE Healthcare Life Sciences).

### Time lapse imaging of cell sorting in cell aggregates

Cell aggregates formed from the intermixing of GFP-labeled WT and unlabeled E-cadherin knockout or unlabeled wild-type ES cells were transferred to CO_2_-independent ES cell media in 100 mm diameter polystyrene culture petri dishes. A thin layer of sterile mineral oil was applied to the top of the media to prevent evaporation. The spheroids were observed under an inverted fluorescence microscope (Nikon TE2000) equipped with a 40X Plan Fluor (NA 0.75, WD 0.72) objective, automatic x-y stage control, z-axis motor, and a temperature-regulated incubation chamber. GFP fluorescence was visualized using a FITC filter (Ex: 450–490 nm). Time-lapse images of consecutive DIC and GFP fluorescence were acquired with a Cascade 650 (Photometrics) monochrome camera (16-bit images) controlled by the MetaVue (Universal Imaging/ MolecularDevices) software every 15 min for 24 to 72 h.

### Quantitation of cell sorting patterns

To quantify the relative positional sorting tendencies of the WT (GFP) cells with respect to the unlabeled co-cultured counterpart - either WT (unlabeled) or E-cad−/− null (unlabeled) cells - two concentric regions of interest (ROI) of equal surface area and together comprising the entire aggregate cross section were analyzed in CorelDRAW X3 (Corel) and ImageJ (NIH) to yield relative, quantitative immunofluorescence intensities. Outer circular ROIs were cast to circumscribe the perimeter of the aggregate cross section; the inner circular ROIs were determined by calculating the dimensions of a circle with an area equal to half of the area of the outer circle. Multiple aggregates were analyzed for the outer and inner distribution of GFP and DAPI signals.

## Supplementary Information

**Additional file 1: Movie 1.** Time-lapse video microscopy of the formation of WT plus WT-GFP ES cell aggregates from 0 to 48 h, with intervals of 20 min.

**Additional file 2: Movie 2.** Time-lapse video microscopy of the formation of E-cadherin null 9j plus WT-GFP ES cell aggregates from 0 to 48 h, with intervals of 20 min.

**Additional file 3: Movie 3.** Time-lapse video microscopy of the formation of E-cadherin null 9j plus WT-GFP ES cell aggregates from 0 to 48 h, with intervals of 20 min. The video shows fusion of two spheroids.

**Additional file 4: Movie 4.** Z-stack confocal sectioning of a representative cell aggregate from E-cadherin (−/−) plus WT-GFP ES, 24-h earlier time course.

**Additional file 5: Movie 5.** Z-stack confocal sectioning of a WT plus WT-GFP ES cell aggregate, 24-h time point.

**Additional file 6: Movie 6.** Z-stack confocal sectioning of an E-cadherin (−/−) plus WT-GFP ES cell aggregate, 48-h time point.

**Additional file 7: Movie 7.** Z-stack confocal sectioning of a WT plus WT-GFP ES cell aggregate, 48-h time point.

**Additional file 8: Movie 8.** Development of E-cadherin (−/−) plus WT-GFP ES cell aggregates, later time course for transition between the two patterns. Imaging started using 24-h pre-formed aggregates, and recording continued for another 24 h.

**Additional file 9: Movie 9.** Development of WT plus WT-GFP ES cell aggregates as a control.

## Data Availability

The datasets used and/or analysed during the current study are available from the corresponding author on reasonable request.

## Abbreviations

bACT-GFP: GFP transgene with beta-actin promoter
DAPI: 4′,6-Diamidino-2-Phenylindole, dihydrochloride
E-Cad: E-cadherin
ES: Embryonic stem
GFP: Green fluorescence protein
kD: Kilo Dalton
RA: Retinoic acid
ROI: Region of interest
WT: Wild-type
